# Dual-doped all-graphene fiber for flexible, high-performance thermoelectric temperature sensing

**DOI:** 10.1038/s41378-026-01412-z

**Published:** 2026-08-25

**Authors:** Feng Han, Qianqian Zhong, Jiacheng Du, Jiajun Hu, Peng An, Yifan Zhao, Kun Zheng, Song Wang, Yaxin Zhang, Dejiang Lu, Chenying Wang, Binbin Jiao, Zhuangde Jiang

**Affiliations:** 1 School of Instrument Science and Technology, Xi’an Jiaotong University, Xi’an, 710049 China; 2 State Key Laboratory for Manufacturing Systems Engineering, Xi’an Jiaotong University, Xi’an, 710049 China; 3 International Joint Laboratory for Micro/Nano Manufacturing and Measurement Technologies, Xi’an Jiaotong University, Xi’an, 710049 China

**Keywords:** Organic-inorganic nanostructures, Organic-inorganic nanostructures

## Abstract

High-performance temperature sensors are critical components for emerging Internet-of-Things and biomedical-electronics platforms. However, simultaneously achieving high sensitivity, mechanical compliance, and user-defined integrability remains a formidable materials-and-device challenge. Here, we report a continuous graphene fiber (GF) thermocouple technology where multiple p–n thermocouples are created in situ along a single, unbroken fiber while preserving its structural integrity. By periodically modulating surface charge-transfer doping with polyethyleneimine (PEI) and FeCl_3_, we formed an array of ten p–n pairs that delivered an exceptional thermocouple sensitivity of 452.32 µV K^−1^. The device retained ~97.8% of its initial sensitivity after 10,000 bending cycles at a 5-mm radius, confirming robustness under repeated mechanical deformation. When deployed on skin, the sensor tracked dynamic body temperature variations with a measurement error of 0.64%, validating its practical value for real-time, non-invasive health monitoring. These results establish all-carbon GF thermocouples as a high-precision and mechanically adaptable temperature-sensing platform for next-generation wearable electronics and personalized healthcare systems.

**Figure:**
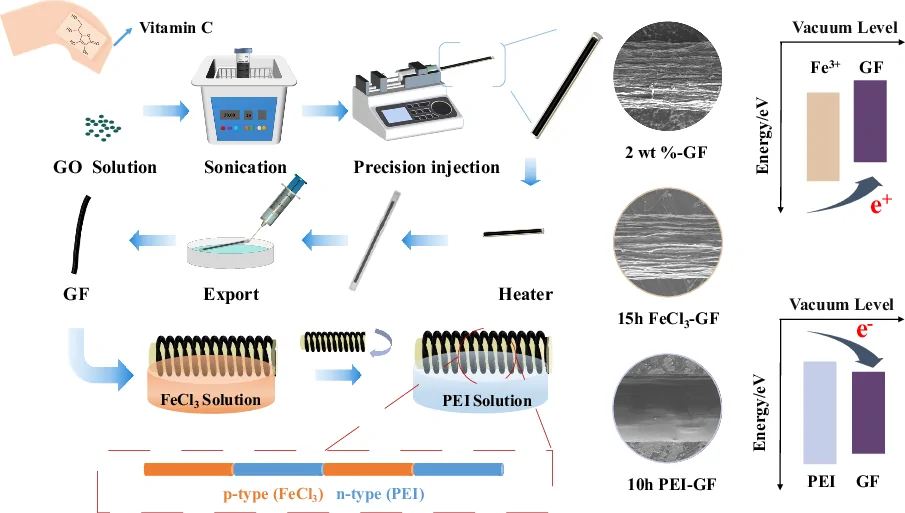

## Introduction

Flexible thermocouple temperature sensors, regarded as core sensing units in flexible electronic systems, provide an essential platform for dynamic thermal management and distributed temperature monitoring^1,2^. Benefiting from their self-powered characteristic of directly converting thermal energy into electrical signals, such sensors have shown broad application prospects in body-temperature management^3,4^, smart bionic skins^5,6^, and personalized medical diagnostics^7,8^. At present, mainstream temperature-sensing technologies—including resistive^9,10^, thermoelectric^11,12^, infrared^13^, and fiber-optic types^14,15^—each exhibits inherent limitations when integrated into flexible electronic systems. Metal-based thermocouples possess wide-temperature responsivity and long-term stability, yet their structural rigidity and macroscale dimensions restrict their integration potential in wearable devices^16,17^. Infrared and fiber-optic techniques enable non-contact detection but suffer from complicated system architectures and high fabrication costs, making large-area, flexible integration difficult to scale. To overcome these bottlenecks, researchers have explored various flexible strategies, such as polymer-composite thermoelectric fibers^13^, printed thermoelectric films^18–20^, and transfer-printed micro-mesh thermocouples^21^. Notably, thermoelectric fibers based on carbon nanomaterials are emerging as ideal candidates for flexible thermoelectric devices due to their intrinsic flexibility and weavability, offering an effective route toward conformal contact with complex curved surfaces^22–24^. However, current flexible thermoelectric materials generally exhibit low sensitivity, insufficient output stability, and performance degradation under repeated mechanical deformation, severely limiting their measurement accuracy and service life. Therefore, developing new composite materials that combine superior thermoelectric conversion efficiency, excellent mechanical robustness, and long-term environmental stability has become an urgent demand for practical applications.

GFs, one-dimensional carbonaceous structures formed by ordered axial stacking and scrolling of two-dimensional graphene sheets^25^, combine superior electrical conductivity, mechanical flexibility, and structural continuity, thus providing a novel platform for flexible sensors^26^. GFs can be prepared on a macroscopic scale via wet spinning^27–29^, confined assembly^30^, or chemical reduction^31^; their microscopic layered architecture enables efficient carrier and heat transport along the fiber axis, leading to a high Seebeck coefficient (α) and improved thermoelectric parameters^4^. Moreover, the highly tunable nature of GFs allows carrier type and concentration to be tuned through ionic doping, offering ample room for constructing multifunctional thermoelectric elements^32,33^.

Herein, we employ the “surface charge-transfer doping” strategy to periodically introduce PEI and FeCl_3_ onto continuous graphene fiber surfaces, accomplishing in situ construction of a p–n thermocouple array and simultaneous interface optimization in one step. By controlling the doping time, the nitrogen and iron states are distributed with a gradient distribution along the radial direction of the fiber, and the interface barrier is adjusted, thereby increasing the thermoelectricity density. The resulting sensor, comprising 10 p–n pairs, delivers an output voltage of 53 mV and a thermocouple sensitivity of 452.32 µV K^−1^ at room temperature; after 10,000 bending cycles under a 5 mm curvature radius, it retains over 97% of its initial stability. When placed on the palm and forearm, the device records skin temperature in real time with a maximum error of 0.64%. This work provides a room-temperature, facile route to high-sensitivity flexible temperature sensors and establishes the material and technological basis for self-powered, high-precision, and weavable temperature-perception networks.

## Results and discussion

**Fig. 1:**
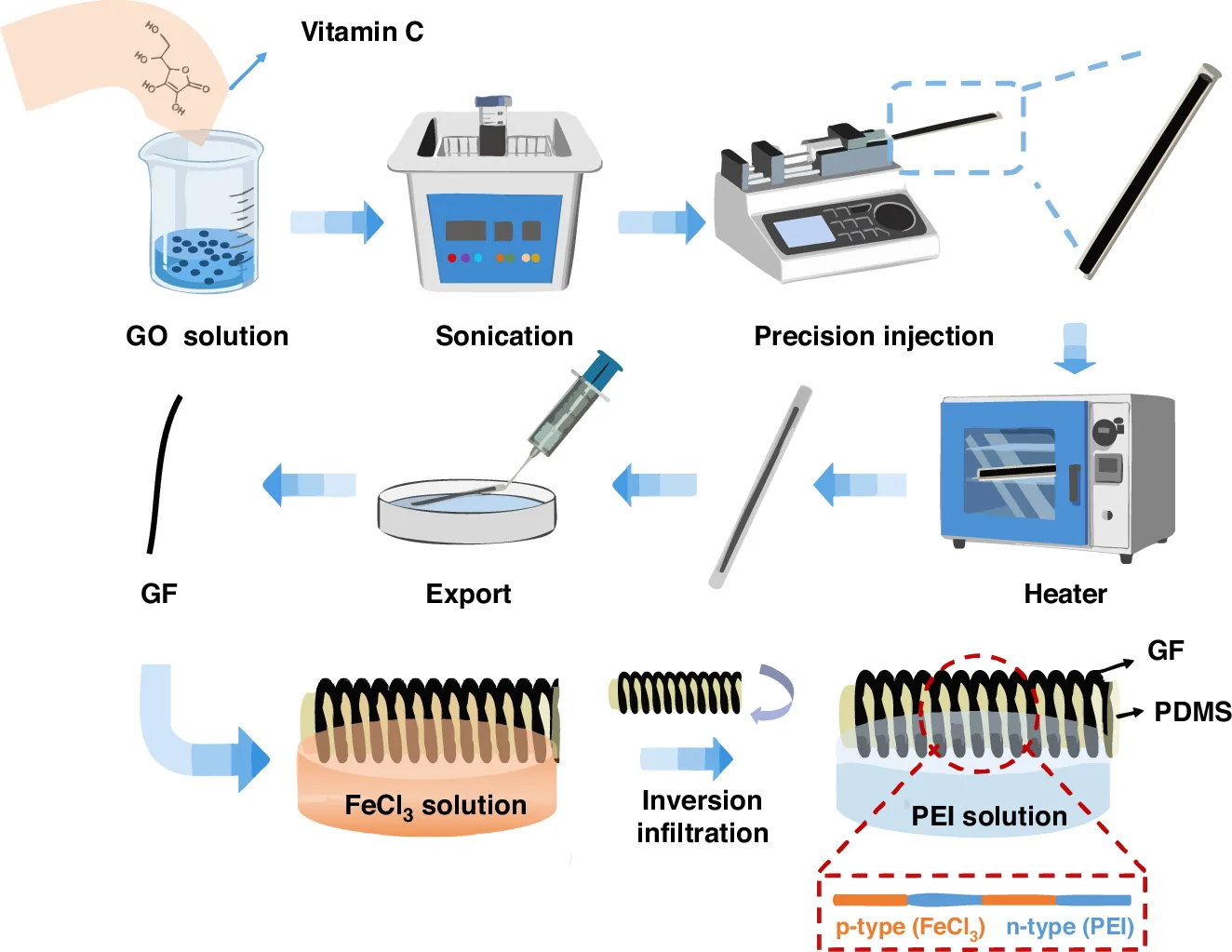
Schematic illustration of the fabrication process of an all-graphene p-n thermocouple by region-speecific doping. GO solution is prepared with Vitamin C, followed by sonication and precision injection into a tubular mold. After thermal reduction, the resultion GF undergress inversion infiltration and selective immersion in FeCl_3_ solution to achieve p-type doping, while the adjacent region is n-type doped with PEI, yielding a GF composite with spatially separated p-n thermocouple

Herein, we report a facile and scalable chemical-reduction hybrid strategy for high-performance graphene fibers, and Fig. 1 illustrates the fabrication process where a syringe pump was employed to inject the graphene oxide precursor into a polytetrafluoroethylene (PTFE) capillary, enabling simultaneous reduction and self-assembly in a sealed cavity to directly yield continuous fibers with tunable length.

**Fig. 2:**
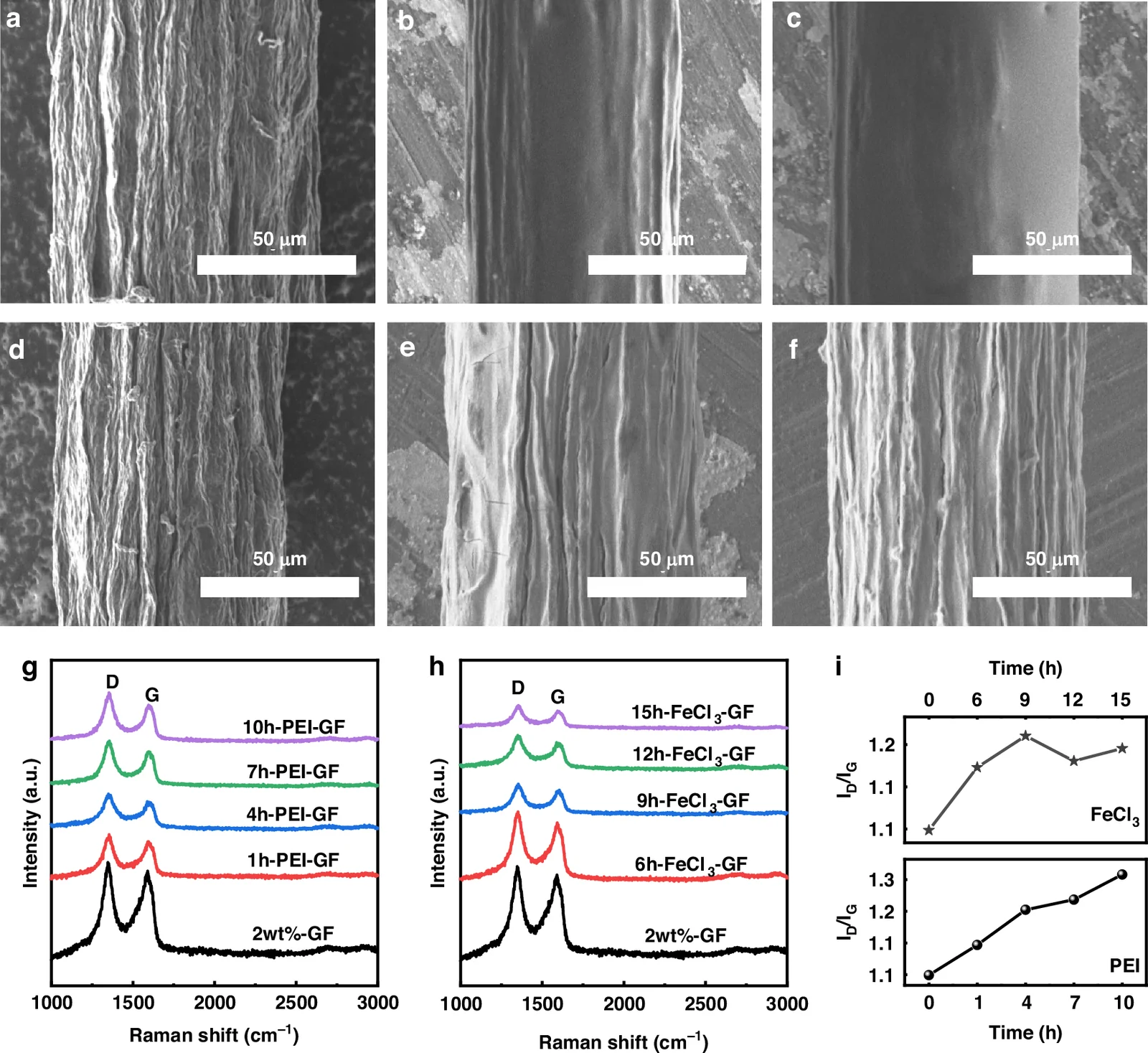
SEM micrographs and Raman spectra of doped graphene fibers. **a**–**c** Surface morphology evolution of pristine GF, 4h-PEI-GF, and 10h-PEI-GF. **d**–**f** Morphology changes in pristine GF, 9h-FeCl_3_-GF, and 15h-FeCl_3_-GF. **g**, **h** Raman spectra showing D and G bands. **i** *I*_D_/*I*_G_ ratios indicating defect evolution with doping time

Figure 2a–f presents representative scanning electron microscopy (SEM) images of 2 wt% vitamin-C-reduced graphene fibers (denoted as 2 wt%-GF) after immersion in PEI or FeCl_3_ solutions for various durations. Under PEI treatment, Fig. 2a–c shows that the hydrophobic graphene lattice strongly adsorbs the organic medium, forming a uniform polymer coating on the outer surface; the fiber diameter increases from 83.3 μm (0 h) to 102.7 μm (1 h) and then gradually decreases with prolonged immersion. Images in Fig. 2d–f illustrate the surface morphology of FeCl_3_-treated fibers: negligible change occurs within 6 h; longer exposure leads to an evident solution sheath, enlarged wrinkles with increased separation, and the emergence of micro-cracks, indicating that extended oxidative doping induces surface reconstruction. Supporting Information Fig. S1 presents high-magnification SEM images of 2 wt%-GF after immersion in PEI or FeCl_3_ solutions for different durations, further demonstrating the effect of immersion time on the surface architecture of 2 wt%-GF. Figure 2g–i displays Raman spectra of PEI-GF and FeCl_3_-GF collected over 1000–3000 cm^−1^. Both sets of fibers exhibit pronounced D (~1350 cm^−1^) and G (~1590 cm^−1^) bands, assigned to structural defects/edge disorder and in-plane sp^2^ graphitic domains, respectively. Peak positions remain essentially unchanged upon either PEI or FeCl_3_ treatment, which evidences preservation of the graphene lattice. For PEI-GF, the *I*_D_/*I*_G_ ratio increases monotonically with doping time: values of 1.07 (0 h), 1.13 (1 h), 1.20 (4 h), 1.22 (7 h), and 1.27 (10 h) are obtained. Literature reports have correlated rising I_D_/I_G_ with reduced average size of sp^2^ domains^34^; additional sp^3^ carbons introduced during polymer functionalization also contribute, confirming successful grafting of PEI chains and effective n-type doping. As-grown GFs are intrinsically p-type due to residual oxygen functionalities. Surface-charge-transfer doping—non-covalent adsorption of electron-donating or -withdrawing species—can invert carrier polarity without disrupting the π-network. The amine-rich PEI serves as an electron donor to afford n-type behavior^35^, whereas FeCl_3_, a strong electron acceptor, enhances hole concentration and thus p-type thermoelectric performance. In FeCl_3_-GF, *I*_D_/*I*_G_ evolves from 1.07 to 1.17, 1.22, 1.18, and 1.20 after 6, 9, 12, and 15 h, respectively. All doped values exceed that of the pristine fiber, but plateau beyond 9 h, indicating that FeCl_3_-induced defect generation saturates quickly.

**Fig. 3:**
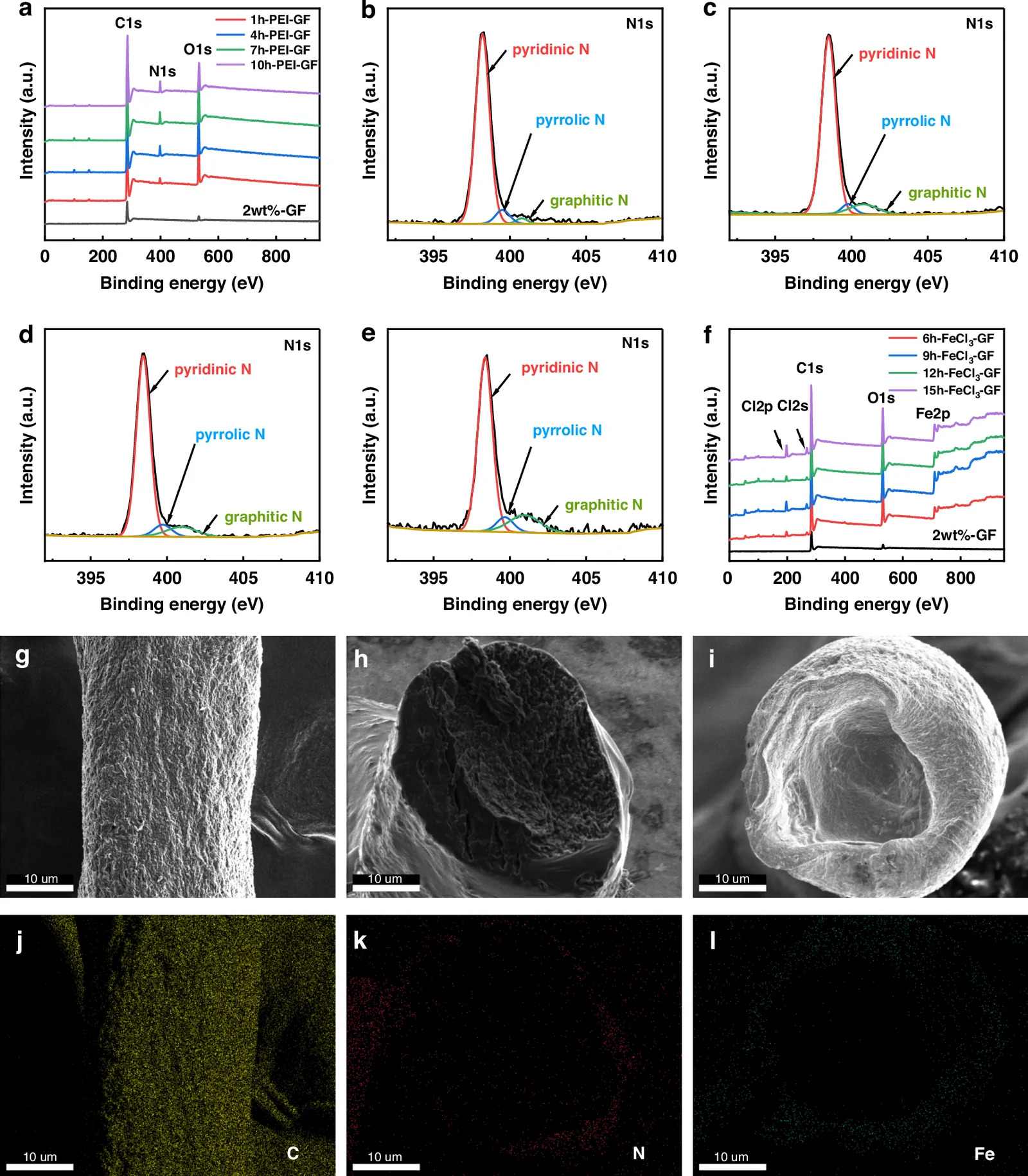
XPS and STEM-EDS characterization of doped graphene fibers. **a** Survey XPS spectra of PEI-GF. **b**–**e** High-resolution N1s spectra showing pyridinic, pyrrolic, and graphitic nitrogen components. **f** Survey spectra of FeCl_3_-GF revealing Fe incorporation. SEM and STEM-EDS images of **g**, **j** pristine GF, **h**, **k** PEI-infiltrated GF, and **i**, **l** FeCl_3_-infiltrated GF. SEM images show the axial (**g**) and radial cross-sectional (**h**, **i**) morphologies

X-ray photoelectron spectroscopy (XPS) was employed to accurately quantify the elemental composition. The survey spectrum of PEI-GF in Fig. 3a exhibits a pronounced N1s signal at 399 eV alongside the intrinsic C1s (284 eV) and O1s (532 eV) peaks of GF, directly confirming the successful incorporation of substantial nitrogen and the formation of a polymer overlayer. Figure S2a–d shows that the high-resolution C1s spectra of 2 wt%-GF can be deconvoluted into four oxygen-containing components, while PEI-GF exhibits three peaks with progressively attenuated C=O and C–O contributions upon prolonged PEI exposure, indicating the reducing action of PEI that eliminates oxygen functionalities and partially restores the π-conjugated network. To establish a correlation between dopant evolution and thermoelectric performance, the nitrogen content and its bonding configurations were systematically analyzed. High-resolution N1s spectra of PEI-GF treated for various durations are presented in Fig. 3b–e; curve-fitting with Advantage software yields nitrogen concentrations of 2.51, 4.41, 5.16 and 5.78 at%, confirming a monotonic increase in nitrogen content with time. N1s deconvolution reveals three nitrogen motifs—pyridinic (398.5 eV), pyrrolic (399.7 eV) and graphitic (400.8 eV), demonstrating successful incorporation into the graphene lattice; their relative fractions can be tuned by doping duration to modulate carrier density and scattering, thereby optimizing the fiber’s thermoelectric performance. Table S1 in the Supporting Information provides quantitative N1s XPS data for PEI-GF at various doping times. Notably, the graphitic nitrogen fraction—which governs n-type doping—peaks at 10.00% at 7 h, correlating with the maximum Seebeck coefficients observed experimentally. As doping time increases, the predominant pathway evolves from pyridinic–pyrrolic coexistence to pyridinic–graphitic synergy, with the graphitic fraction reaching a maximum at 7 h. At the atomic scale, pyridinic and pyrrolic nitrogens preferentially occupy edge sites, whereas graphitic nitrogen substitutes carbon atoms within the basal plane. Electronically, pyridinic and pyrrolic nitrogens withdraw π-electrons from graphene, thereby imparting p-type character; in contrast, graphitic nitrogen donates its excess valence electron into the graphene π-conjugated system, forming occupied ${\pi }^{* }$ donor states above the Dirac point that shift the Fermi level upward^36,37^. This n-type charge injection breaks the sublattice symmetry and drives the opening of a tunable bandgap, the magnitude of which scales with the graphitic nitrogen concentration. Figure 3f presents survey spectra of FeCl_3_-GF recorded at various doping times; the five distinct peaks—Cl2p (197 eV), Cl2s (268 eV), C1s (284 eV), O1s (532 eV) and Fe2p (710 eV)—confirm the successful incorporation of iron into the graphene fibers, which evidences effective modification of the pristine GF. The high-resolution Fe2p spectra in Fig. S3a–d exhibit doublets at 710.4 eV (Fe2p_3/2_) and 725.2 eV (Fe2p_1/2_), whose binding energies and line shapes are characteristic of Fe^3+^. Table S2 shows that the Fe content reaches a maximum of 3.82% at 9 h, indicating the optimal doping time for achieving the highest Fe^3+^ incorporation. Scanning transmission electron microscopy–energy-dispersive X-ray spectroscopy (STEM-EDS) imaging was employed to directly visualize the elemental distribution within the graphene fibers. Figure 3g, j presents the axial SEM image and corresponding carbon elemental mapping of pristine GF, establishing the uniform carbon framework throughout the fiber as the structural baseline. Figure 3h, k displays the radial cross-section of PEI-infiltrated GF and its nitrogen distribution, revealing pronounced N enrichment at the fiber periphery with gradual attenuation toward the core. Figure 3i, l illustrates the radial cross-section of FeCl_3_-infiltrated GF and its iron distribution, showing analogous Fe segregation at the outer region with inward depletion. Both dopant elements exhibit distinct edge-concentrated profiles that depart markedly from the homogeneous C distribution, preliminarily indicating the formation of radial concentration gradients. More quantitatively, Fig. S4 elucidates the time-dependent evolution of this gradient architecture. For PEI-N, infiltration progresses from 1 h to 7 h with progressively intensified edge-enriched characteristics as shown in Fig. S4a–c, establishing an optimal radial concentration gradient at 7 h; similarly, FeCl_3_-Fe demonstrates escalating peripheral enrichment from 6 h to 9 h as shown in Fig. S4e, f, reaching peak gradient distribution at 9 h. Notably, these optimal infiltration times (7 h for PEI-N and 9 h for FeCl_3_-Fe) coincide precisely with the peak thermoelectric performance observed in subsequent measurements. This progressive concentration of dopants at the fiber periphery constructs continuous interfacial energy barriers at heterogeneous junctions. According to the theoretical model for sequential interfacial systems, an appropriate energy barrier height enables selective scattering of low-energy carriers while preferential transmission of high-energy carriers—this energy filtering effect elevates the mean carrier energy and substantially enhances the Seebeck coefficient^38^. Consequently, carrier transport in optimally gradient-doped GFs is dominated by high-energy electrons, leading to optimized thermoelectric performance. However, prolonged infiltration disrupts this favorable scenario: at 10 h for PEI-N (Fig. S4d) and 12–15 h for FeCl_3_-Fe (Fig. S4g, h), both elements progressively diffuse toward the fiber interior, evolving from edge-enriched to quasi-uniform distribution. This diminution of the concentration gradient reduces the effective barrier height and compromises its continuity; as dictated by energy filtering theory, an excessively low or discontinuous barrier offers negligible selectivity for carrier filtering, thereby invalidating the energy filtering effect and constraining Seebeck coefficient enhancement. Thus, the thermoelectric performance is critically governed by the delicate balance between gradient establishment and preservation—sufficient infiltration time is requisite to construct effective interfacial barriers, yet excessive duration erodes the very architectural feature that enables carrier energy filtering.

**Fig. 4:**
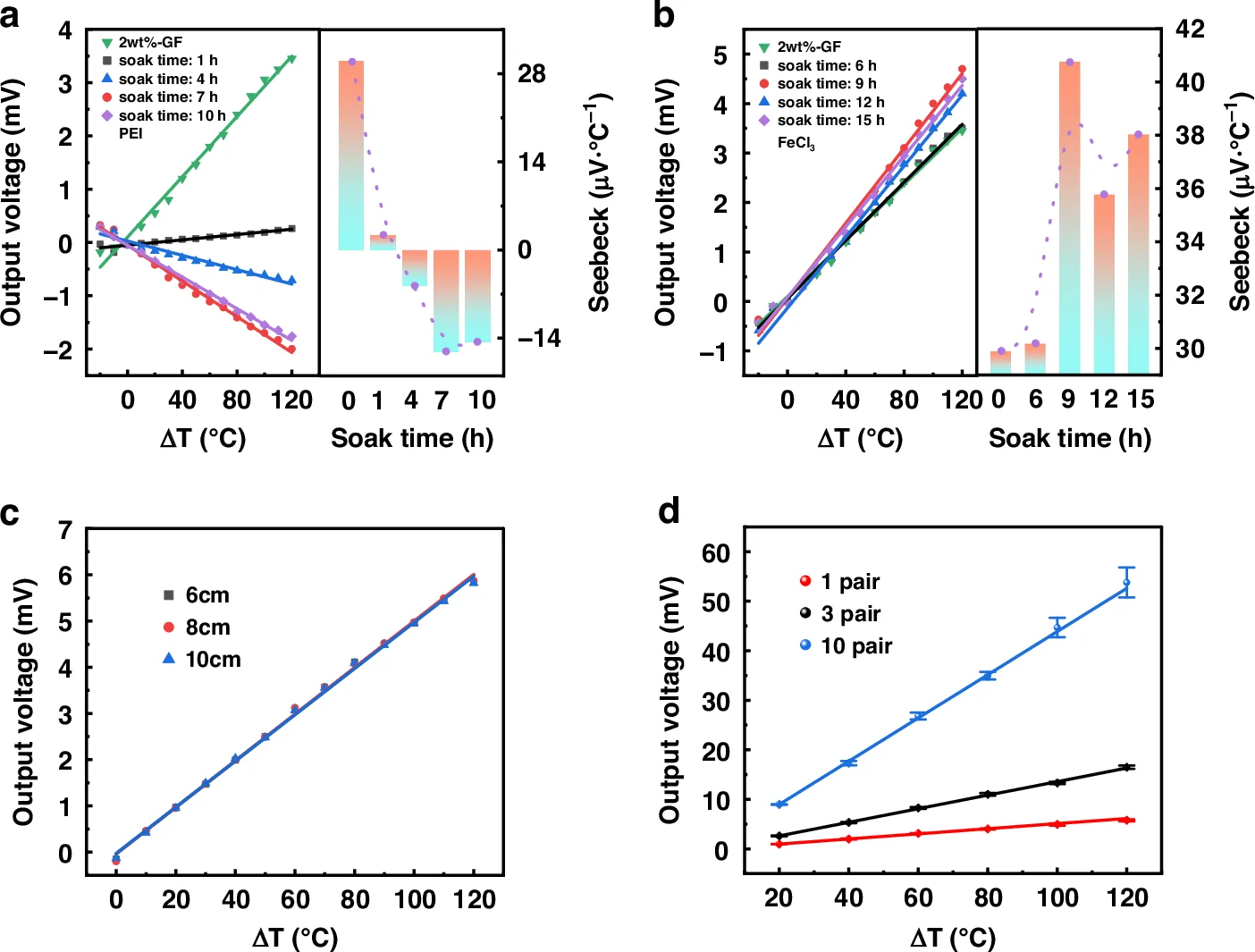
Thermoelectric and NTC characteristics of graphene fiber sensors. **a**, **b** Output voltage and Seebeck coefficients of PEI-GF and FeCl_3_-GF thermocouples. **c** Length-independent thermocouple sensitivity. **d** Linear scalability with p–n couple number

To quantitatively evaluate the influence of PEI and FeCl_3_ doping on the thermoelectric performance of graphene fibers, fiber-Pt thermocouples were fabricated, and their output characteristics were systematically measured. As shown in Fig. 4a, b, all devices exhibit a good linear thermoelectric voltage response within the −20–120 °C range, presenting a stable temperature-dependent Seebeck coefficient. This indicates good applicability of the thermocouples in various scenarios (e.g., cold-chain monitoring, fever diagnosis). As shown in Fig. 4a, extending the PEI doping time to 4 h or longer induces a distinct polarity reversal in the output voltage, confirming the transition from p-type to n-type conduction. The maximum thermoelectric output is attained at 7 h, which coincides with the highest graphitic nitrogen content revealed by XPS, underscoring the pivotal role of graphitic nitrogen in enhancing n-type thermoelectric performance. When the PEI doping time is 4 h or longer, the output voltage polarity is reversed, confirming a p-type to n-type transition; the maximum output was achieved at 7 h, where XPS results show the highest graphitic nitrogen content, highlighting the key role of graphitic nitrogen in enhancing n-type thermoelectric performance. For FeCl_3_ doping, the peak output (+4.7 mV) was reached at 9 h, consistent with the strongest Fe signal in XPS, indicating that Fe species significantly enhance p-type thermoelectric properties, as illustrated in Fig. 4b. Table S3 in the Supporting Information summarizes the output fitting parameters and Seebeck coefficient for all thermocouple sensors. Linear fitting yields high correlation coefficients (*R*^2^ > 0.99) for all samples, with the maximum Seebeck coefficients reaching +40.74 µV K^−1^ for the FeCl_3_-doped (9 h) sample and −16.12 µV K^−1^ for the PEI-doped (7 h) sample, confirming the carrier-type inversion between p-type (FeCl_3_) and n-type (PEI) doping.

The sensitivity (S) of the thermoelectric temperature detector is defined as the rate of change of output voltage with respect to temperature difference, expressed by the equation: $s=\frac{{dv}}{d\triangle T}={n}_{n}{\alpha }_{n}+{n}_{p}{\alpha }_{p}$, where ${\alpha }_{n}$ and ${\alpha }_{P}$ represent the absolute Seebeck coefficients of the n-type and p-type fibers, respectively, and ${n}_{n}$ and ${n}_{P}$ denote the respective quantities of n-type and p-type fibers integrated within the detector. Benefiting from the tailorable length of graphene composite fibers, sensors with thermocouple lengths of 3, 4, and 5 cm (corresponding to total fiber lengths of 6, 8, and 10 cm) were prepared. Figure 4c shows that the output voltage maintains an excellent linear relationship with the temperature difference across all lengths; due to the good uniformity of the thermoelements, the thermocouple sensitivity remains stable at ≈50 µV K^−1^ with minimal fluctuation. At the same temperature difference, the output voltages of devices with different lengths are nearly identical, demonstrating that sensitivity is independent of length and fully verifying the scalability of the continuously integrated thermocouple sensor in the length dimension. Devices containing 1, 3, and 10 p–n thermocouples were further assembled, with all p-type and n-type segments directly interconnected through node contacts without any external metal wires. To ensure statistical reliability, three independently fabricated devices were characterized for each configuration. Figure 4d presents the output voltage as a function of temperature difference, with error bars indicating the standard deviation across the three devices. The thermocouple sensitivities, extracted from the linear slopes, are 50.16 ± 1.5, 138.18 ± 3.2, and 452.32 ± 9.8 µV K^−1^ (mean ± standard deviation, *n* = 3) for the single-couple, 3-couple, and 10-couple devices, respectively—corresponding to 4×, 11×, and 36× enhancements over the 12.5 µV K^−1^ reported for a single-junction graphene fiber thermocouple obtained by dual-doping chemistry^4^. The linear increase in sensitivity with the number of p–n pairs is consistently reproduced across all independent devices, with relative standard deviations below 3%, confirming the robustness of the in-situ doping and interconnection process. As shown in Fig. S5, all graphene fiber samples exhibit typical negative temperature coefficient (NTC) semiconductor behavior, i.e., resistance decreases exponentially with increasing temperature. This is consistent with the crumpled graphene thermistor behavior reported by Yan et al.^38^.

**Fig. 5:**
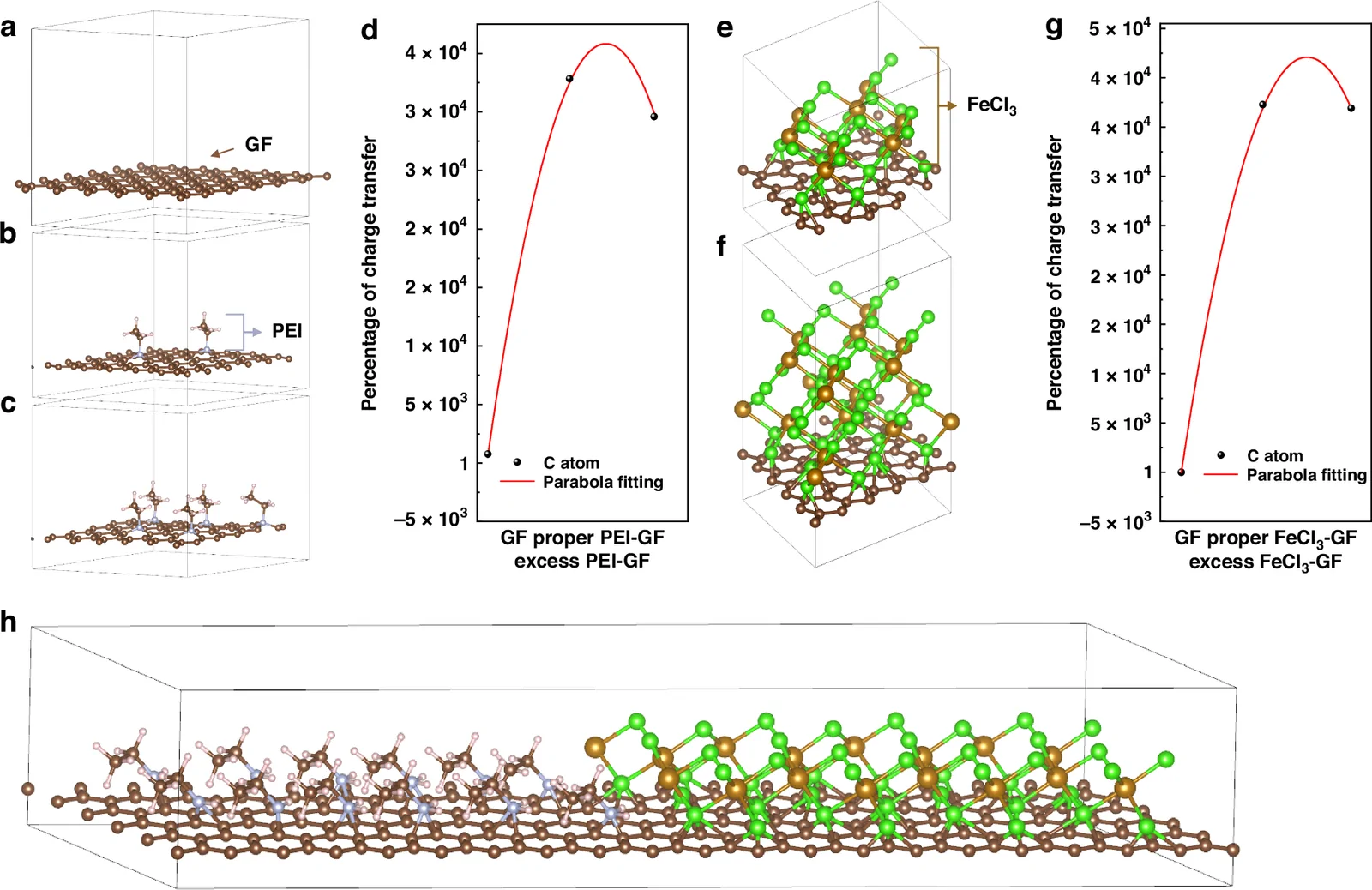
DFT models and charge transfer analysis. **a**–**c**, **e**, **f**, **h** Atomic structure models of pristine graphene film (GF), polyethyleneimine-functionalized graphene (PEI-GF), ferric chloride-functionalized graphene (FeCl_3_-GF), and PEI/FeCl_3_-GF p-n heterojunction, where brown, pink, blue, green, and orange spheres represent carbon, hydrogen, nitrogen, chlorine, and iron atoms, respectively. **d**, **g** Bader charge analysis quantitatively revealing the charge transfer efficiency between dopants and graphene and its concentration dependence

To elucidate the charge transfer efficiency between PEI/FeCl_3_ and graphene, DFT models with varying doping concentrations were established (Fig. 5a–c, e, f). As shown in Fig. 5d, optimal PEI loading elevates the charge transfer percentage of graphene carbon atoms to ~3.3 × 10^4^%, a 4-order increase, while Fig. 5g demonstrates that appropriate FeCl_3_ loading reaches ~3.8 × 10^4^%, a 4-order enhancement. This efficient n-type and p-type charge injection originates from graphitic nitrogen serving as electron donor and Fe^3+^ as electron acceptor, respectively^39,40^. PEI overloading reduces the percentage to ~2.9 × 10^4^% with ~12% decay, but retains nearly 4 orders of enhancement. FeCl_3_ overloading merely decreases to ~3.7 × 10^4^% with ~2.6% decay, preserving 4 orders of improvement. Excessive doping induces efficiency decay, yet the optimization remains robust. The deformation potential calculations reveal exceptionally high carrier effective masses in the PEI/FeCl_3_-GF p-n heterojunction as shown in Fig. 5h. The effective masses are extracted from the band curvature near the valence-band maximum (VBM) and conduction-band minimum (CBM) via second-order derivative fitting, i.e., $\frac{1}{m}=\frac{1}{{\hslash }^{2}}\frac{{\partial }^{2}E(k)}{\partial {k}^{2}}$, yielding ${{\rm{m}}}_{e}^{* }=1.87{{\rm{m}}}_{0}$ for electrons and ${{\rm{m}}}_{h}^{* }=2.75{{\rm{m}}}_{0}$ for holes. These values originate from the delocalized $\pi /{\pi }^{* }$ transport states in the vicinity of the Fermi level, whose wave functions are extended throughout the graphene layer and dominate the charge transport. The deep-level flat bands associated with localized Fe 3d states, located at −1.5 to −0.5 eV with wave functions tightly confined around Fe atoms, do not participate in extended transport and are therefore excluded from the effective-mass evaluation. The obtained effective-mass values are consistent with first-principles studies on asymmetrically dual-doped graphene, where inversion-symmetry breaking by acceptor–donor pairs transform the linear Dirac dispersion into a parabolic band, thereby increasing the carrier effective mass from nearly zero to a few. According to the relation $\alpha \propto {m}^{* }$, this mass enhancement significantly increases the Seebeck coefficient. Simultaneously, the corresponding carrier mobilities are calculated via deformation-potential theory with explicit inclusion of interfacial charged-impurity scattering—the dominant scattering mechanism in chemically doped graphene arising from charged dopants at the graphene–dopant interface^41^. These mobility values agree well with experimental reports for PEI-doped graphene and heavily FeCl_3_-intercalated graphene^42,43^. Based on the Drude model ($\sigma ={ne}\mu$), even with moderate mobilities of ~50 cm^2 ^V^−1^ S^−1^ and typical charge-carrier densities of 10^12^–10^13^ cm^−2^ for heavily doped graphene, the sheet conductivity can readily reach ~0.005 S/sq, which is fully compatible with the good electrical conductivity demonstrated in our devices. The p–n junction interface potential barrier between the graphitic nitrogen-doped region and the Fe^3+^- doped region, in collaboration with the enhanced effective masses, generates an energy-filtering effect, selectively blocking low-energy holes and forcing high-energy electrons to dominate the transport, thereby further amplifying the Seebeck coefficient.

**Fig. 6:**
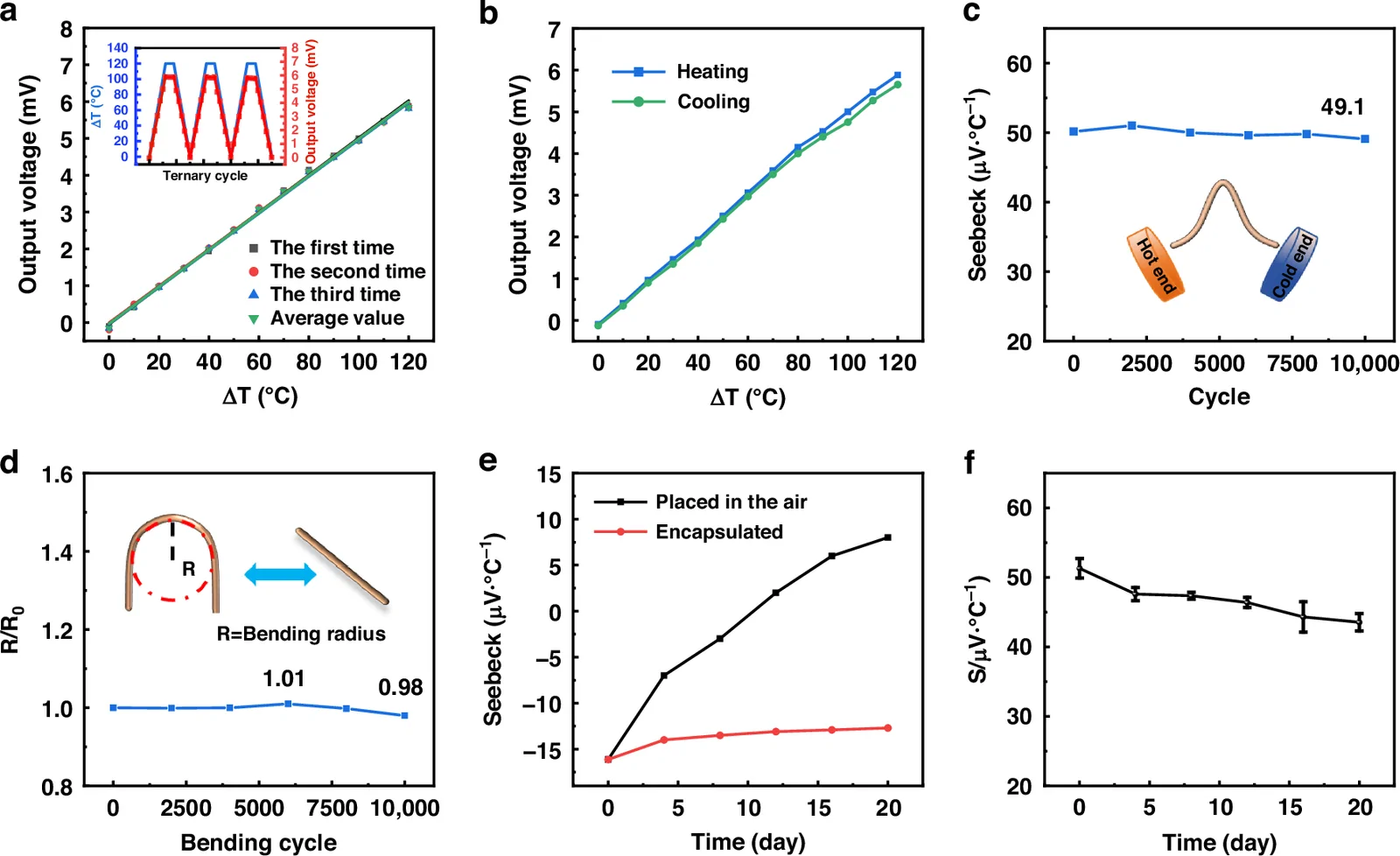
Sensor stability and durability tests. **a** Triplicate heating repeatability with fitted curves. **b** Hysteresis error (4.27%). **c**, **d** Mechanical stability over 10,000 bending cycles for sensitivity and resistance. **e** Long-term Seebeck evolution with/without encapsulation. **f** 20-day sensitivity retention of encapsulated device (87.6%)

The deviation between output values obtained from multiple measurements under the same input quantity can be employed to characterize the repeatability of a sensor. For temperature sensors, repeatability serves as a critical metric for evaluating their long-term operational stability. To achieve optimal sensor thermocouple sensitivity, graphene fibers doped with PEI for 7 h and FeCl_3_ for 9 h were selected to construct a thermocouple temperature sensor. The thermoelectric performance of the as-fabricated PEI-GF/FeCl_3_-GF thermocouple temperature sensor was evaluated utilizing a static calibration methodology. Triplicate repetitive heating tests were conducted on the sensor over the temperature range of 0–120 °C, with calibration performed at 10 °C intervals to establish the correlation between output thermoelectric voltage and temperature difference. To intuitively elucidate the output characteristics of the sensor, we subjected the experimental data to least-squares fitting to establish a mathematical relationship between temperature and thermoelectric voltage, yielding the fitted curves for the triplicate heating repeatability tests of the thermocouple temperature sensor, which are depicted in Fig. 6a. The thermoelectric potential output of the PEI-GF/FeCl_3_-GF thermocouple temperature sensor maintains a good linear function relationship with the temperature difference between the cold and hot ends. When the temperature difference is 120 °C, the maximum output thermoelectric potential is 5.85 mV. The fitting *R*^2^ of the output curve for the three-time temperature rise repeatability test data of the thermocouple temperature sensor is all greater than 99.7%, and the sensitivity of the sensor can reach 50.23 µV K^−1^. The three cycles of heating and cooling of the sample were tested using the static calibration test device of the thermocouple. The maximum temperature difference of the thermocouple was 120 °C and the holding time was 10 min. The changes in the output thermoelectric potential of the thermocouple during the three static tests and the holding process were recorded, and the static test and holding characteristic curves of the temperature sensor were obtained, as shown in the inset of Fig. 6a. It can be seen that the curves were highly consistent in multiple tests, and the maximum output voltage during heating was at the same level. The temperature difference and output voltage during multiple heating processes were both linear relationships, and the repeated test data were almost completely overlapping. The same was true for the cooling process. This proves that the thermocouple temperature sensor has good thermal response. The least square method was used to fit, and *R*^2^ was greater than 0.99, indicating a good linear relationship. Thus, it can be seen that the thermocouple has good cycle stability and repeatability.

Under identical operating conditions, the agreement between forward and reverse strokes is quantified by the hysteresis characteristic, calculated as:

1 $${\gamma }_{H}=\frac{{\Delta H}_{\max }}{{Y}_{{FS}}}$$

where ${\gamma }_{H}$ is hysteresis error of the sensor; ${\varDelta H}_{\max }$ is maximum deviation between forward and reverse outputs at the same input; ${Y}_{{FS}}$ is full-scale output.

After a full 0–120 °C heating–cooling cycle, the hysteresis characteristic is presented in Fig. 6b. Using Eq. (1), the maximum hysteresis error is calculated to be 4.27%. This deviation originates primarily from intrinsic material defects and structural imperfections; signal lag of the reference K-type thermocouple also contributes. Future efforts will focus on compositional optimization, microstructure engineering, and fabrication protocol improvements to minimize hysteresis. It should be noted that the 4.27% hysteresis was characterized under a wide-range dynamic thermal cycle (0–120 °C), where the lag arises from the combined thermal inertia of the sensor components and the reference K-type thermocouple. In practical wearable applications such as skin-temperature monitoring, the sensor operates under quasi-static conditions with a narrow temperature window (Δ*T* ≈ 6.6 °C). By allowing the sensor to reach thermal equilibrium before readout and employing an in-situ calibration protocol based on repeated placement–removal cycles, the hysteresis-induced deviation is effectively suppressed^44^. To evaluate operational durability under repeated bending, a PI-integrated PEI-GF/FeCl_3_-GF thermocouple temperature sensor was subjected to cyclic flex tests. The 6-cm-long fiber was bent 90° with a 5 mm curvature radius at 1 mm s^−^¹ on a linear stage for 10,000 cycles. The resistance increased marginally to ~101% at 6000 cycles, then gradually declined, declining to ~98% at 10,000 cycles, resulting in a net change of less than 2%. To assess the structural integrity of the thermoelectric fibers under mechanical fatigue, post-bending SEM characterization was conducted on GF, PEI-GF, and FeCl_3_-GF after 10,000 bending cycles, as presented in Fig. S6. For the pristine GF, the low- and high-magnification images before bending, as shown in Fig. S6a_1_, a_2_, reveal a densely packed surface with characteristic longitudinal graphitic textures, whereas after 10,000 cycles (Fig. S6a_3_, a_4_) slight surface loosening and minor fibril peeling appear at the outer curvature, yet no penetrating cracks or interlayer delamination are observed, confirming the inherent flexibility of the continuous graphene backbone. For PEI-GF the pre-bending images (Fig. S6b_1_, b_2_) show a uniform surface morphology, and after 10,000 cycles (Fig. S6b_3_, b_4_) the fiber surface remains intact without discernible cracks or structural disruption, which is attributed to the inherent flexibility of the PEI molecular chains and their robust interfacial adhesion via amine–π interactions. In contrast, for FeCl_3_-GF, the p-type doping relies on FeCl_3_, which tends to form brittle ionic clusters on the fiber surface; the pre-bending images (Fig. S6c_1_, c_2_) display a relatively smooth surface, whereas after cyclic bending (Fig. S6c_3_, c_4_) localized micro-cracks emerge within these superficial FeCl_3_-rich regions due to the brittle nature of the dopant aggregates under cyclic tensile/compressive strain. Notably, these micro-cracks remain confined to the outermost doped surface and do not propagate through the fiber cross-section, indicating that the underlying graphene core maintains its electrical and structural integrity. Collectively, these results demonstrate that all three fiber types preserve their continuous conductive pathways after prolonged bending, and the PI encapsulation layer further mitigates any risk of environmental ingress through these superficial defects. The stable electrical and thermoelectric responses demonstrate the sensor’s capability for long-term operation under dynamic bending conditions. When PEI-GF is directly exposed to ambient air, incomplete surface coverage allows adsorption of water and oxygen, leading to rapid degradation of n-type characteristics and a reversion to p-type behavior. Consequently, PEI-doped graphene can be regarded as a composite that supports both electrons and holes, and the total thermoelectric sensitivity is expressed as^45^:

2 $$S=\frac{{S}_{e}{\sigma }_{e}+{S}_{h}{\sigma }_{h}}{\sigma }$$

Where *S* is total sensitivity; ${S}_{e}$ is electron-derived component (negative); ${S}_{h}$ is hole-derived component (positive); ${\sigma }_{e}$, ${\sigma }_{h}$ are electron and hole conductivities; σ is total conductivity.

Under PEI doping, electrons dominate, yielding S < 0. Air exposure increases the hole population, reducing ${|S|}$. After 12 days, the sensitivity switches sign to +2 µV K^−1^, indicating hole majority transport. Encapsulating PEI-GF with a Polyimide (PI) film retards this process: the sensitivity changed slowly from −16.12 to −13.1 µV K^−1^ over 12 days and stabilized at ≈−12.7 µV K^−1^ thereafter, confirming that the barrier layer effectively blocks oxygen adsorption and preserves n-type behavior as shown in Fig. 6e. To evaluate the long-term stability of the PI-encapsulated PEI-GF/FeCl_3_-GF thermocouple, three independently fabricated devices were tightly sealed with PI film and stored in an environmental chamber maintained at 25 ± 1 °C and 60 ± 5% relative humidity under indoor ambient lighting with a 12 h/12 h light/dark cycle. The Seebeck coefficient was measured at days 0, 4, 8, 12, 16, and 20. As shown in Fig. 6f, the sensitivity decreased from an initial value of 51.31 ± 1.40 µV K^−1^ to 43.55 ± 1.23 µV K^−1^ after 20 days, corresponding to a retention of 84.9 ± 2.4% (mean ± standard deviation, *n* = 3). The shaded error band represents the standard deviation across the three independent devices, confirming the batch-to-batch reproducibility of the encapsulation and fabrication process. After careful analysis, we attribute the 12% performance decay of the current PI encapsulation over 20 days to two primary coupled failure mechanisms. First, the inherent hygroscopicity of the imide ring structure in polyimide films facilitates moisture absorption from sweat or high-humidity environments, leading to film swelling and the propagation of micro-cracks that progressively compromise the encapsulation barrier integrity. Second, once moisture breaches this barrier, the active doping layers are directly degraded: polyethyleneimine exhibits strong hydrophilicity and water solubility, rendering it susceptible to swelling, migration, and leaching under moist or electrolyte-rich conditions, whereas iron(III) chloride is highly hygroscopic and readily undergoes hydrolysis, causing a sharp decline in p-type doping efficiency; the synergistic degradation of both dopants disrupts the carefully engineered charge-transfer balance at the p–n junctions, resulting in a concurrent deterioration of the Seebeck coefficient and electrical conductivity^46^. To address these limitations, it is promising to achieve a rigid–flexible gradient encapsulation through a fully elastomeric potting strategy coupling an outer superhydrophobic PDMS/nano-SiO_2_ composite coating with an inner PI support layer, thereby further enhancing the long-term durability and thermoelectric performance stability of flexible thermoelectric temperature sensors in harsh environments. In this integrated architecture, the hydrophobically modified PDMS/SiO_2_ surface constructs micro/nano hierarchical roughness to block moisture permeation, the PDMS elastomer matrix accommodates mechanical stress through its inherent compliance while completely isolating the device from sweat and electrolytes, and the inner PI film provides sustained structural support and electrical insulation, thus synergistically suppressing the coupled failure behaviors of hygroscopic swelling, dopant hydrolysis/leaching, and interfacial delamination. To extend operational lifetime, more robust encapsulation strategies should be pursued in future work.

**Fig. 7:**
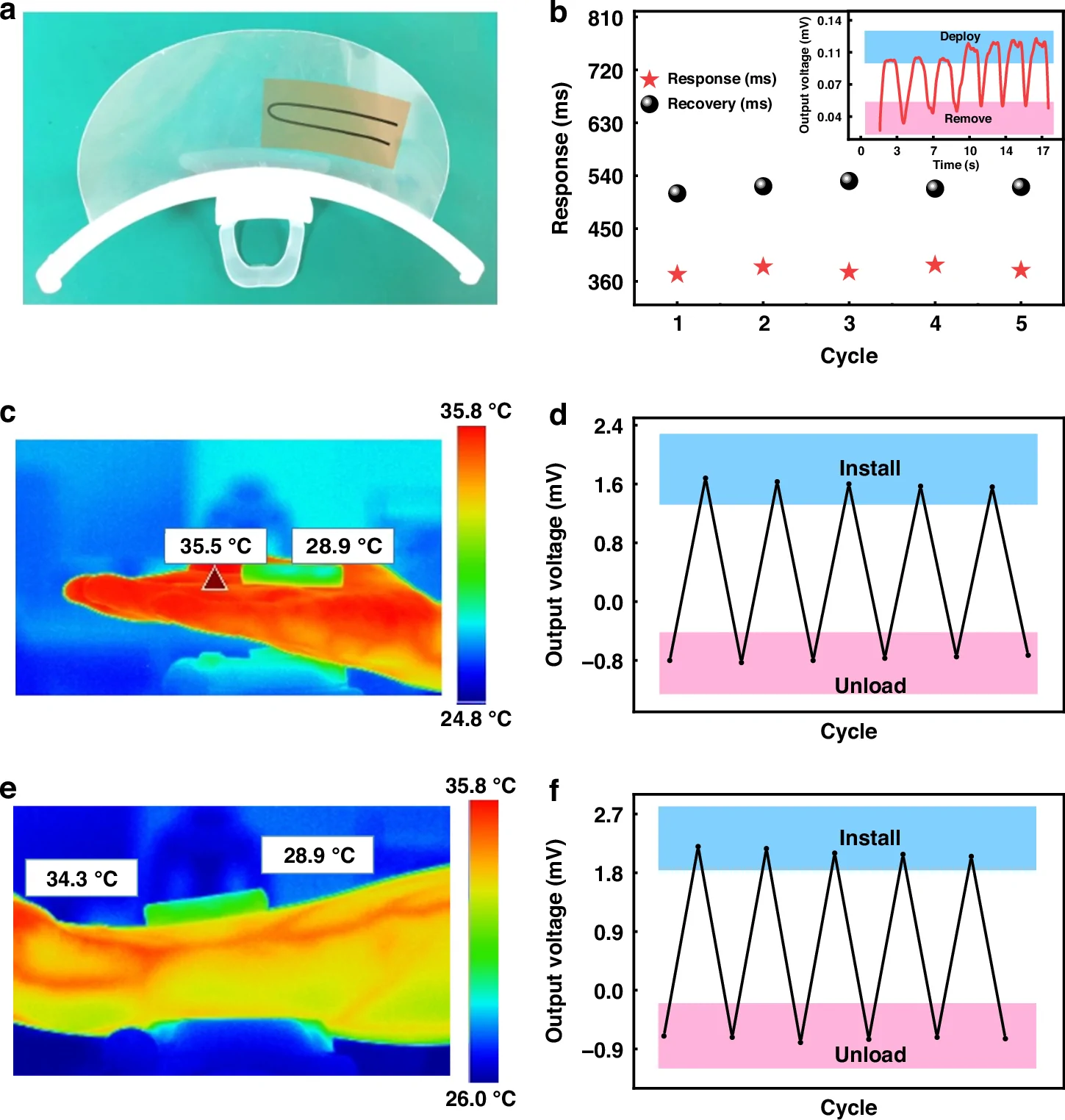
The practical applications of the thermocouple sensor. **a** Schematic illustration of the thermocouple attached to the inner surface of a face mask. **b** Response and recovery times extracted from consecutive respiration cycles (cycles 3–7) using the 10–90% threshold criterion. Inset: thermoelectric voltage signal generated during periodic breathing. **c**, **e** Infrared thermographs of the 10-couple thermocouple for temperature detection on the human palm and forearm, respectively. **d**, **f** Cyclic output voltage results when the sensor is placed on the palm and forearm, respectively

To contextualize the observed Seebeck enhancement, Table S4 in the Supporting Information summarizes key parameters of recently reported graphene and carbon nanotube-based thermoelectrics. Existing devices in this category typically exhibit Seebeck coefficients below 240 µV K^−1^ and further suffer from limited bending durability and high fabrication cost. In contrast, our optimized 10-pair PEI-GF/FeCl_3_-GF p–n junction device achieves a record high Seebeck coefficient of 452.32 µV K^−1^, a value that represents nearly double the best reported literature performance. Beyond this exceptional thermoelectric output, the device simultaneously delivers outstanding bending durability, maintains sensitivity retention exceeding 97%, and preserves baseline production cost. Respiratory rate is a vital physiological parameter. A thermocouple-based, non-invasive and dynamic respiration monitor can provide continuous, real-time breathing data for intra-operative care, post-operative analgesia, post-anesthesia recovery and critical-care management. Here, a PEI-GF/FeCl_3_-GF thermocouple was taped inside a face mask with the hot junction positioned centrally near the mouth/nose to sense the breath flow and the cold junction exposed to ambient air (Fig. 7a). During breathing, periodic hot-air pulses (26.1–37.3 °C) heated the hot junction, generating a temperature difference (Δ*T*) that followed the respiratory rhythm; the resulting thermoelectric voltage indicated a respiratory period of approximately 2.2 s (Fig. 7b inset). To further evaluate the dynamic sensing behavior, the transient output voltage was analyzed during the periodic application and removal of thermal stimuli. The response and recovery times were determined using a unified 10–90% threshold criterion, where the response time corresponds to the signal increase from 10 to 90% of the signal amplitude, and the recovery time corresponds to the decrease from 90 to 10%. Based on five consecutive respiration cycles (cycles 3–7), the sensor exhibited a response time of approximately 380 ms and a recovery time of approximately 520 ms, with only minor cycle-to-cycle fluctuations, demonstrating good repeatability and stable real-time physiological monitoring capability. These dynamic characteristics compare favorably with those of commercial flexible temperature sensors, including graphene-based thermistors and metal-film sensors. The response/recovery times of commercial flexible temperature sensors are shown in Table S5. Continuous body-temperature monitoring is essential for health management. Ten pairs of PEI-GF/FeCl_3_-GF thermocouples were integrated on a polydimethylsiloxane (PDMS) strip and attached to the palm: the skin-contact region served as the hot junction, air-exposed region=cold junction, with temperatures calibrated by an Infrared (IR) camera (Fig. 7c). The sensor underwent repeated attachment/removal cycles, which were recorded as shown in Fig. 7d. For the palm skin at 35.5 °C and the cold junction at 28.9 °C (Δ*T* = 6.6 °C), the sensor output was 2.05–2.20 mV (±0.15 mV), corresponding to Δ*T* = 6.50–6.83 °C and a calculated palm temperature of 35.4–5.73 °C, which deviated by ≤0.23 °C from the IR value (error 0.64%). The forearm measurement showed an IR temperature of 34.3 °C with a cold junction of 28.9 °C (Δ*T* = 5.4 °C). The sensor output of 1.56–1.68 mV (±0.12 mV) corresponds to Δ*T* = 5.43–5.69 °C and a forearm temperature of 34.33–34.59 °C, with a maximum deviation of 0.29 °C (0.84% error), mainly due to local heating from sensor-skin friction. The exceptional accuracy of the measurements demonstrates its capability for precise temperature mapping on the skin surface and validates its application prospects in next-generation flexible electronics and continuous physiological monitoring.

## Conclusions

In summary, we have fabricated high-performance all-carbon flexible thermoelectric materials and devices by a periodic surface charge-transfer doping of individual graphene fibers with PEI and FeCl_3_ solutions to create alternating p- and n-type segments. Systematic modulation of the doping duration enabled quantitative elucidation of the effects of nitrogen- and iron-containing species on the fiber microstructure, surface morphology, and thermoelectric properties. On this basis, a PEI-GF/FeCl_3_-GF flexible thermocouple temperature sensor was constructed and comprehensively characterized in terms of thermocouple sensitivity, bending robustness, repeatability, and long-term stability. The device exhibited a thermocouple sensitivity of 452.32 µV K⁻¹ and a body-temperature measurement error as low as 0.64%, while retaining excellent mechanical flexibility and scalability. Real-time respiration and skin-temperature monitoring capabilities were experimentally validated. These results establish the all-carbon graphene fiber as a promising candidate for high-performance flexible thermoelectrics and offer a new route toward wearable, self-powered, and high-precision personal health-monitoring systems.

## Experimental section

### Materials and reagents

GFs were synthesized via a confined hydrothermal assembly route assisted by vitamin C–mediated chemical reduction. In brief, 5 mL of a 12 mg mL^−1^ graphene oxide (GO) aqueous dispersion was sonicated for 30 min to eliminate aggregates. Subsequently, vitamin C (VC, 2 wt% with respect to GO) was added as a green reducing agent, and the mixture was stirred vigorously to ensure homogeneity. After 5 min of static equilibration, the suspension was degassed under vacuum to remove trapped air bubbles, guaranteeing dense and uniform fiber formation. The degassed solution was then gently injected into a PTFE capillary; both ends were sealed, and the assembly was maintained at 150 °C for 2 h, enabling GO sheets to self-assemble into fibrillar architectures within the confined channel. Owing to the presence of VC, the reaction temperature was deliberately lowered from the conventional 180 °C used for pure GO hydrothermal processing to prevent structural degradation. Upon completion, the resultant fibers were extracted from the PTFE tube and dried under ambient conditions, which afforded graphene oxide fibers and VC-reduced graphene fibers, respectively.

### Construction of periodic p–n type structure

To engineer a graphene-fiber thermocouple sensor with periodically alternating p–n segments, 2 wt%-GF exhibiting the highest Seebeck coefficients was selected as the active fiber. A cylindrical PDMS support was first fabricated: PDMS prepolymer and curing agent were mixed at a 10:1 mass ratio, degassed under vacuum until bubble-free, poured into a cylindrical PTFE mold, degassed again, and cured at 60 °C for 3 h to yield a dense PDMS substrate as shown in Fig. S7. The 2 wt%-GF was then uniformly wound around the PDMS cylinder, and its two ends were subjected to spatially selective doping: one tip was immersed in 30 wt% PEI solution for n-type doping, while the opposite tip was immersed in 45 wt% FeCl_3_ solution for p-type doping. Doping durations were systematically varied (1, 4, 7, 10 h for PEI; 6, 9, 12, 15 h for FeCl_3_) to optimize carrier type and concentration. After doping, the fiber was dried on a 50 °C hot plate for 15 min. This protocol creates axially periodic and well-ordered p-type and n-type domains along a single continuous graphene fiber, affording a graphene-fiber thermocouple temperature sensor with tailored thermoelectric response. Although the current static soaking protocol is effective for laboratory-scale demonstration, it can be translated into a continuous roll-to-roll (R2R) process by unwinding the graphene fiber through sequential modular stations: selective dopant deposition via microfluidic patterning or aerosol-jet printing, in-line rapid curing to suppress interdiffusion, and rotary masking to ensure opposite-side PEI and FeCl_3_ coating. Sharp p–n boundaries can be preserved by employing segmented gas-shielding masks or microfluidic channel isolation between the n-type and p-type deposition zones, thereby minimizing axial dopant diffusion. This R2R-compatible strategy has been demonstrated for continuous carbon-nanotube fiber electronics, confirming the scalability of the present approach^47^.

### Characterization and measurements

Morphological characterization of graphene fibers was performed using a field-emission scanning electron microscope (FESEM, SU-8010, Hitachi, Tokyo, Japan). Raman spectra were acquired with a laser Raman spectrometer (HR800, Horiba Jobin Yvon, Paris, France). Surface chemistry and electronic structure were analyzed by X-ray photoelectron spectroscopy (XPS, ESCALAB Xi+, Thermo Fisher, Waltham, MA, USA). Electrical conductivity was measured with a digital source meter (2450, Keithley, Beaverton, OR, USA), while output voltage was recorded by a data acquisition unit (LR8450, HIOKI, Nagano, Japan). The temperature difference between the hot and cold junctions of the sensor was determined from infrared thermal images captured by an IR camera (Fotric 225 s).

## Supplementary information

Supplementary information

## Data Availability

The data that support the findings of this study are available from the corresponding author upon reasonable request.
