# Supplementary material for: Dual-doped all-graphene fiber for flexible, high-performance thermoelectric temperature sensing
Source: Microsyst Nanoeng. 2026 Aug 25;12:305. doi: 10.1038/s41378-026-01412-z (PMC13503711; doi:10.1038/s41378-026-01412-z)
Supplement: Supplementary file 1 [file 41378_2026_1412_MOESM1_ESM.docx]

Supporting Information

**Dual-Doped All-Graphene Fiber for Flexible, High-Performance Thermoelectric Temperature Sensing^[1]^**

Feng Han,^1,2,3^ Qianqian Zhong,^1,2,3^ Jiacheng Du,^1^ Jiajun Hu,^1^ Peng An,^1^ Yifan Zhao,^1,2,3^ Kun Zheng,^2^ Song Wang,^1,2,3^ Yaxin Zhang,^2,3^ Dejiang Lu,^1,2,*^ Chenying Wang,^1,2,3,*^ Binbin Jiao,^1,2,3^ and Zhuangde Jiang,^2,3^

**Keywords:** Graphene fiber; Dual-doping; Temperature sensor; Flexible materials

Figure S1. High-magnification SEM micrographs of 2 wt%-GF after immersion in PEI or FeCl_3_ solutions. (a–e) Surface morphology evolution of fibers treated with PEI for 0 h (a), 1 h (b), 4 h (c), 7 h (d), and 10 h (e), showing progressive polymer coating formation. (f–j) Surface changes of fibers treated with FeCl_3_ for 0 h (f), 6 h (g), 9 h (h), 12 h (i), and 15 h (j), illustrating oxidative doping-induced surface reconstruction.

Figure S2. High-resolution XPS C1s spectra of doped graphene fibers. (a–d) Deconvoluted C1s envelopes showing three characteristic components: C–C (~284.8 eV), C=N/C–O (~286 eV), and C–N/C=O (~288 eV). The progressive attenuation of oxygen-related peaks and emergence of nitrogen-associated bonds confirm successful surface functionalization and the concurrent reducing action of PEI on graphene oxide.

Table S1 Quantitative N1s XPS data for PEI-GF at various doping times

| Sample | 1h-PEI-GF | 4h-PEI-GF | 7h-PEI-GF | 10h-PEI-GF |
| --- | --- | --- | --- | --- |
| Pyridinic Nitrogen /% | 76.30% | 86.20% | 83.33% | 89.28% |
| Pyrrolic Nitrogen /% | 16.07% | 4.32% | 6.67% | 2.68% |
| Graphitic Nitrogen /% | 7.63% | 9.48% | 10.00% | 8.04% |

Figure S3. High-resolution Fe2p XPS spectra of FeCl_3_-doped graphene fibers. (a–d) Fe2p spectra of 6h-FeCl_3_-GF (a), 9h-FeCl_3_-GF (b), 12h-FeCl_3_-GF (c), and 15h-FeCl_3_-GF (d), exhibiting characteristic doublets at 710.4 eV (Fe2p_3/2_) and 725.2 eV (Fe2p_1/2_) confirming the presence of Fe^3+^ species.

Table S2 Relative Fe content of FeCl_3_-GF as a function of doping time

| Sample | 6h-FeCl_3_-GF | 9h-FeCl_3_-GF | 12h-FeCl_3_-GF | 15h-FeCl_3_-GF |  |
| --- | --- | --- | --- | --- | --- |
| Fe /% | 2.18% | 3.82% | 3.20% | 3.70% |  |
|  | | | | | |
| Figure S4. STEM-EDS elemental mapping of GF cross-sections at different infiltration times. (a-d) Distribution of N from PEI at infiltration times of 1, 4, 7, and 10 h, respectively, with 7 h being the optimal time exhibiting distinct edge-enriched gradient characteristics; (e-h) distribution of Fe from FeCl_3_ at infiltration times of 6, 9, 12, and 15 h, respectively, with 9 h being the optimal time showing the most favorable edge-doped gradient architecture. | | | | | |

Table S3 Output fitting parameters of the thermocouple sensors

| Sample | V~ΔT/μV | Seebeck /μV ℃^-1^ | R^2^ |
| --- | --- | --- | --- |
| 2wt%-GF/Pt | V=-23.30+29.90ΔT | 29.90 | 0.997 |
| 1h-PEI-GF/Pt | V=-40.99+2.38ΔT | 2.38 | 0.991 |
| 4h-PEI-GF/Pt | V=-40.99-5.71ΔT | −5.71 | 0.993 |
| 7h-PEI-GF/Pt | V=-52.86-16.12ΔT | −16.12 | 0.993 |
| 10h-PEI-GF/Pt | V=-74.84-14.60ΔT | −14.60 | 0.995 |
| 6h-FeCl_3_-GF/Pt | V=-5.05+30.19ΔT | 30.19 | 0.997 |
| 9h-FeCl_3_-GF/Pt | V=-15.72+40.74ΔT | 40.74 | 0.997 |
| 12h-FeCl_3_-GF/Pt | V=-118.35+35.78ΔT | 35.78 | 0.997 |
| 15h-FeCl_3_-GF/Pt | V=-98.90+38.03ΔT | 38.03 | 0.998 |

Figure S5. NTC semiconductor behavior of all samples

Figure S6. SEM characterization of GF, PEI-GF, and FeCl_3_-GF fibers before and after 10^4^ bending cycles (radius: 5 mm; speed: 1 mm/s). (a) Pristine GF: (a_1_, a_2_) before bending at low and high magnification; (a_3_, a_4_) after bending showing slight surface loosening. (b) PEI-GF: (b_1_, b_2_) before bending; (b_3_, b_4_) after bending showing stable morphology. (c) FeCl_3_-GF: (c_1_, c_2_) before bending; (c_3_, c_4_) after bending showing localized micro-cracks. Scale bars: 100 μm for low-magnification images (×350) and 50 μm for high-magnification images (×1.00k–1.10k).

**Simulation method of thermocouple temperature sensor**

**Computational Methods:**

All calculations were performed using first-principles calculations based on spin-polarized density functional theory (DFT), implemented via the Vienna Ab initio Simulation Package (VASP). The electron exchange-correlation interactions were described using the Perdew-Burke-Ernzerhof (PBE) functional within the generalized gradient approximation (GGA). The plane-wave basis set was truncated at 500 eV to ensure computational accuracy for energy and forces. Brillouin zone integration was performed using the Γ-centered Monkhorst-Pack method with a 3×3×1 k-point mesh, which was verified to achieve full convergence of total energies and electronic structure properties. The DFT-D3 method (based on Grimme's empirical correction scheme) was employed to account for van der Waals weak interactions. Atomic positions were optimized using the conjugate gradient algorithm, with an energy convergence criterion of 5×10^-6^ eV for electronic self-consistent iterations and a force convergence threshold of 0.01 eV/Å for ionic relaxation, thereby ensuring that both electronic ground states and geometric structures were fully relaxed to equilibrium.

**Carrier Mobility Calculations:**

Carrier mobilities were calculated based on the deformation potential theory for long-wavelength acoustic phonon scattering. For two-dimensional materials, the carrier mobility (μ) is expressed as:

$$\mu_{2D}=\frac{e\hbar^{3}C_{2D}}{k_{B}Tm^{*}m_{d}E_{1}^{2}}$$

where ，$e$ is the elementary charge, $\hbar$ is the reduced Planck constant, $k_{B}$ is the Boltzmann constant, and $T$ is the temperature. Key material parameters include: $m^{*}$ is the effective mass of carriers (electrons or holes), $m_{d}$ is the average effective mass (density-of-states effective mass); $E_{l}$ is the deformation potential constant, defined as the first derivative of the carrier valley energy (conduction band minimum or valence band maximum) with respect to strain ($E_{l} = dE/d\varepsilon$), which is obtained by performing band structure calculations on systems subjected to small uniaxial strains (±0.5% to ±1%) followed by linear fitting. $C_{2D}$ is the 2D elastic modulus (in-plane stiffness), determined by calculating the system's elastic modulus and subsequent data post-processing.

After obtaining $m^{*}$、$E_{l}$ and $C_{2D}$ from the aforementioned first-principles calculations, the theoretical intrinsic mobility can be derived by substituting these values into the equation.

**Bader Charge Analysis:**

Bader charge analysis based on first-principles calculations was performed using the VASP package. Upon completion of structural optimization and static self-consistent calculations, files containing the total charge density and valence electron charge density were exported. Subsequently, the Bader Charge Analysis program developed by the Henkelman group was utilized to acquire the Bader charges and atomic magnetic moments (if spin polarization is involved) for each atom via topological partitioning of the charge density, thereby enabling quantitative characterization of charge transfer and bonding characteristics between atoms.

Table. S4. Graphene and carbon nanotube-based sensors and their material properties

| **Ref** | **Material System** | **p–n Pairs** | **Seebeck Coefficient (µV K^-1^)** | **Bending Cycles** | **Sensitivity Retention** | **Costing** |
| --- | --- | --- | --- | --- | --- | --- |
| [1] | Graphene fiber / Pt wire | 8 | 200 | 1 000 | 95.70 % | High |
| [2] | 3D-printed Graphene/ Silicate Bulk | – | 20.14 | – | – | High |
| [3] | Expandable Graphite/ Cement Bulk | – | 180.5 | – | – | Moderate |
| [4] | Graphene Fiber Seamless Kno | 1 | 12.5 | 1 000 | >95 % | Moderate |
| [5] | Graphene/ Ecoflex Extruded Filament | – | 37.2 | 1 000 | ≈100 % | Low |
| [6] | SWCNT Fiber Module | 8 | ≈111 | – | – | Moderate |
| [7] | UHMWPE/ GO Bulk Tablet | – | 235 | – | – | Low |
| [8] | Graphene/ PEDOT: PSS Composite Fiber | 3 | 17.4 | – | – | Low |
| [9] | Single-Material U-Shaped Graphene | 1 | 39 | – | – | High |
| [10] | Bromine-Doped Graphene Fiber | - | 50 | – | – | Moderate |
| [11] | PEDOT: PSS/SWCNTs Composite Film | 6 | 70.1 | – | – | Moderate |
| [12] | FeCl_3_/PEI-Doped CNT Yarn | 60 | 81 | – | – | High |
| [13] | PEI-Doped SWNT Continuous Network | 3 | 140 | – | >95 % | High |
| [14] | p-type: F4TCNQ-doped CNT web  n-type: BV-doped annealed CNT web | 10 | 233.7 | 50 | - | Moderate |
| This work | p-type: GF-FeCl_3_  n-type: GF-PEI | 10 | 452.32 | 10000 | ＞97% | Low |

Table S5 Literature Comparison of Response/Recovery Time of Flexible Temperature Sensors

| **Sensor type** | **Active material** | **Response time** | **Recovery time** |
| --- | --- | --- | --- |
| Commercial flexible thermistor[OMEGA] | Hermetically sealed flexible thermistor | ~3.75 s | Not specified |
| Commercial flexible thermistor[muRata] | NTC thermistor with flexible leads | ~3 s | ~3 s |
| Commercial flexible sensor[15] | Commercial flexible thermistor | ~62 s | ~120 s |
| Graphene-based thermistor[16] | Laser-scribed rGO | ~1.2 s | Not specified |
| Graphene/PDMS sensor[17] | Graphene/PDMS composite | ~22.4 s | ~31.0 s |
| Metal film sensor[18] | Silver (Ag) film thermistor | Several 10 s | Not specified |
| This work | Graphene | ~380 ms | 520 ms |

Figure S7. Photographs of a thermocouple temperature sensor. (a) Graphene fiber wound on a PTFE mold. (b) Stress applied to both ends of the PTFE mold. (c) Sensor placed on the palm.

**References**

[1] C. Wang, Y. Zhang, F. Han, Z. Jiang, Flexible thermoelectric type temperature sensors based on graphene fibers, Micromachines (Basel) 14 (2023) 1853,
    https://doi.org/10.3390/mi14101853.
[2] K. Songa, J. Liu, P. He, Q. Zhang. 3D printing graphene lattice enabled smart silicate composites with piezoresistive and thermoelectric effects, Carbon, 234 (2025) 120026, [https://doi.org/10.1016/j.carbon.2025.120026](https://spis.hnlat.com/scholar/redirect?url=https%3A%2F%2Fdoi.org%2F10.1016%2Fj.carbon.2025.120026).
[3] J. Wei, L. Sheng, E. Lv, Z. Jiang, W. Gao, Y. Guo, H. Zhang, X. Li Significant improvement in thermoelectric properties of carbon-reinforced cementitious composites achieved by regulating the graphitization degree of expanded graphite, ACS Applied Materials Interfaces 16 (2024) 35381-35389, [https://doi.org/10.1021/acsami.4c08270](https://spis.hnlat.com/scholar/redirect?url=https%3A%2F%2Fdoi.org%2F10.1021%2Facsami.4c08270).
[4] S. Kim, S. Lim, M.H. Jeong, W. Kim, S. Baik, J.W. Suk, Flexible thermocouple using a thermoelectric graphene fiber with a seamless junction, Journal of Materials Science & Technology 172 (2024) 15-22, https://doi.org/10.1016/j.jmst.2023.05.078.
[5] H. Li, D. Zhang, C. Wang, Y. Hao, Y. Zhang, Y. Li, P. Bao, H. Wu, 3D extruded graphene thermoelectric threads for self-powered oral health monitoring, Small 19 (2023) e2300908, [https://doi.org/10.1002/smll.202300908](https://spis.hnlat.com/scholar/redirect?url=https%3A%2F%2Fdoi.org%2F10.1002%2Fsmll.202300908).
[6] X.G. Xia, Q. Zhang, W.B. Zhou, Z.J. Xiao, Y.C. Wang, W.Y. Zhou, W. Xi, Highly flexible and excellent performance continuous carbon nanotube fibrous thermoelectric modules for diversified applications, Chinese Physics B 30 (2021) 078801, https://doi.org/10.1088/1674-1056/abff33.
[7] A. Irfan, M.N. Mehmood, M.S. Mehmood, A. Aziz, M.A. Baluch, M. Rizwan, T. Yasin, A potential approach to enhance the seebeck coefficient of uhmwpe by using the graphene oxide, Non-Metallic Material Science 2 (2020) 21, https://doi.org/10.30564/omms.v2i2.2601
[8] J. Liu, G. Liu, J. Xu, C. Liu, W. Zhou, P. Liu, G. Nie, X. Duan, F. Jiang, Graphene/polymer hybrid fiber with enhanced fracture elongation for thermoelectric energy harvesting, ACS Applied Energy Materials 3 (2020) 6165-6171, [https://doi.org/10.1021/acsaem.0c00001](https://spis.hnlat.com/scholar/redirect?url=https%3A%2F%2Fdoi.org%2F10.1021%2Facsaem.0c00001).
[9] A. Harzheim, F. Könemann, B. Gotsmann, H. van der Zant, P. Gehring. Single‐material graphene thermocouples, Advanced Functional Materials 30 (2020) 2000547, [https://doi.org/10.1002/adfm.20200057](https://doi.org/10.1002/adfm.202000574).
[10] W. Ma, Y. Liu, S. Yan, T. Miao, S. Shi, Z. Xu, X Zhang, C. Gao, Chemically doped macroscopic graphene fibers with significantly enhanced thermoelectric properties, Nano Research 11 (2017) 741-750, [https:// doi.org/10.1007/s12274-017-1683-3](https://www.sci-hub.vg/10.1007/s12274-017-1683-3).
[11] H. Song, Y. Qiu, Y. Wang, K. Cai, D. Li, Y. Deng, J. He, Polymer/carbon nanotube composite materials for flexible thermoelectric power generator, Composites Science and Technology 153 (2017) 71-83, https://doi.org/10.1016/j.compscitech.2017.10.006.
[12] J. Choi, Y. Jung, S.J. Yang, J.Y. Oh, J. Oh, K. Jo, J.G. Son, S.E. Moon, C.R. Park, H. Kim, Flexible and robust thermoelectric generators based on all-carbon nanotube yarn without metal electrodes, ACS Nano 11 (2017) 7608-7614, https://doi.org/10.1021/acsnano.7b01771.
[13] W. Zhou, Q. Fan, Q. Zhang, L. Cai, K. Li, X. Gu, F. Yang, N. Zhang, Y. Wang, H. Liu, W. Zhou, S. Xie, High-performance and compact-designed flexible thermoelectric modules enabled by a reticulate carbon nanotube architecture, Nature Communications 8 (2017) 14886, https://doi.org/10.1038/ncomms14886.
[14] C.J. An, Y.H. Kang, H. Song, Y. Jeong, S.Y. Cho. High-performance flexible thermoelectric generator by control of electronic structure of directly spun carbon nanotube webs with various molecular dopants, Journal of Materials Chemistry A 5 (2017) 15631-15639, [https://doi.org/10.1039/C7TA02509B](https://spis.hnlat.com/scholar/redirect?url=https%3A%2F%2Fdoi.org%2F10.1039%2FC7TA02509B).
[15] S. Das, K. Thiyagarajan, S. Kodagoda, A. Krishnan, M. Bhattacharjee, A novel tactile sensing skin for surface perception, IEEE Sensors Journal 25 (2025) 23155-23162,
     https://doi.org/10.1109/JSEN.2025.3565586
[16] G.Y. Liu, Q.L. Tan, H.R. Kou, L. Zhang, J.Q. Wang, A flexible temperature sensor based on reduced graphene oxide for robot skin used in internet of things, Sensors 18 (2018) 1400,
     https://doi.org/10.3390/s18051400
[17] L. Liu, Y.Y. Dou, J.H. Wang, Y. Zhao, W.W. Kong, Recent advances in flexible temperature sensors: materials, mechanism, fabrication, and applications, Advanced Science 11 (2024) 2405003, <https://doi.org/10.1002/advs.202405003>
[18] R. Liu, L. He, M. Cao, Z. Sun, R. Zhu, Y. Li, Flexible temperature sensors, Frontiers in Chemistry 9 (2021) 539678, <https://doi.org/10.3389/fchem.2021.539678>

1. ^*^Corresponding author. Dejiang Lu: [djlu@xjtu.edu.cn](mailto:djlu@xjtu.edu.cn); Chenying Wang: [wangchenying@mail.xjtu.edu.cn](mailto:wangchenying@mail.xjtu.edu.cn)

   1 School of Instrument Science and Technology, Xi’an Jiaotong University, Xi’an 710049, China.

   2 State Key Laboratory for Manufacturing Systems Engineering, Xi’an Jiaotong University, Xi’an 710049, China.

   3 International Joint Laboratory for Micro/Nano Manufacturing and Measurement Technologies, Xi’an Jiaotong University, Xi’an 710049, China.
